# Uncoupling protein 2 and signal transducer and activator of transcription 1 are targets of human papillomavirus oncoproteins and may be prognostic markers for cervical cancer development

**DOI:** 10.3389/fonc.2025.1689058

**Published:** 2025-11-24

**Authors:** Mariana Carmezim Beldi, Fabiane Cristina Colunna, Noely Paula Lorenzi, Jordy Alexander Lasso Larco, Laura Sichero, Edmund Chada Baracat, Vanesca de Souza Lino, Enrique Boccardo, Giana Mota, Luisa Lina Villa, Maricy Tacla, Marcia Farina Kamilos, Maria Luiza Nogueira Dias Genta, Ana Paula Lepique

**Affiliations:** 1 Immunology Department, Instituto de Ciências Biomédicas, Universidade de São Paulo, São Paulo, Brazil; 2 Gynecology Department, Hospital Universitário, Universidade de São Paulo, São Paulo, Brazil; 3 Instituto do Câncer do Estado de Sao Paulo ICESP, Hospital das Clínicas da Faculdade de Medicina da Universidade de São Paulo FMUSP HC, São Paulo, Brazil; 4 Comprehensive Center for Precision Oncology, Universidade de São Paulo, São Paulo, Brazil; 5 Gynecology Department, Faculdade de Medicina, Universidade de São Paulo, São Paulo, Brazil; 6 Microbiology Department, Instituto de Ciências Biomédicas, Universidade de São Paulo, São Paulo, Brazil; 7 Department of Radiology and Oncology, Faculdade de Medicina, Universidade de São Paulo, São Paulo, Brazil; 8 Gynecology Department, Hospital das Clínicas, Universidade de São Paulo, São Paulo, Brazil; 9 Hospital Heliópolis, São Paulo, Brazil

**Keywords:** uncoupling protein 2, signal transducer and transcription activator 1, human papillomavirus, oxidative stress, inflammation

## Abstract

**Introduction:**

Cervical cancer, predominantly caused by persistent high-risk Human Papillomavirus (HPV) infection, remains a significant public health issue in developing countries. Identifying prognostic markers for patients with precursor lesions could improve clinical management and outcomes.

**Methods:**

We performed a comparative gene expression analysis between low- and high-grade cervical intraepithelial lesions, focusing on genes associated with inflammation and oxidative stress. STAT1 (Signal Transducer and Activator of Transcription 1) and UCP2 (Uncoupling Protein 2), known for their roles in other cancers, were selected for further validation. Immunodetection techniques were applied in both monolayer and organotypic cultures to assess the regulation of these proteins by HPV oncoproteins. Additionally, immunohistochemistry was conducted on two patient groups: those with precursor lesions plus cancer, and those with cervical cancer only.

**Results:**

In cultured HaCaT cells transduced with E6/E7 HPV oncogenes, expression levels of STAT1 and UCP2 changed, especially in organotypic cultures. In patient samples, both UCP2 and STAT1 levels increased with the severity of cervical precursor lesions, and their expressions showed a strong correlation. Notably, nuclear STAT1 expression, indicative of protein activation, was rare in cancer samples but correlated with poor prognosis. In contrast, positive UCP2 expression was linked to improved survival rates and reduced recurrence.

**Discussion:**

Our findings demonstrate that HPV oncoproteins modulate the expression of UCP2 and STAT1. These proteins may serve as valuable prognostic markers for patients with precursor cervical lesions, and UCP2 expression, in particular, could be beneficial for predicting outcomes in cervical cancer patients.

## 1 Introduction

The etiologic factor of cervical cancer is persistent infection with high oncogenic risk Human Papillomavirus (HPV) (1, 2). Despite the introduction of HPV prophylactic vaccines, cervical cancer remains a significant public health burden, especially in low-income countries (3). Cervical cancer usually takes years or decades to develop after HPV infection. During this period, there is a stepwise neoplastic progression that includes the development of precursor lesions (4), from low- to high-grade (Low- and High-grade Squamous Intraepithelial Lesions, LSIL and HSIL, respectively). During this process, E6 and E7 oncoproteins immortalize and transform epithelial cells (5) through a complex process that involves changes in cell signaling, activation of telomerase activity, accumulation of genetic defects, and immune evasion (6). From a histopathological perspective, LSIL is characterized by a well-differentiated but abnormal epithelium, with alterations restricted to the lower third of the tissue. Typically, LSIL represents a productive infection that may regress within 2 years post-infection. In fact, data from the literature indicate that only 0.03% of LSIL cases progress to cervical cancer (7). Conversely, most women diagnosed with HSIL will progress to cervical cancer in relatively short periods if left untreated. These lesions display abnormal mitotic figures and undifferentiated cells throughout the epithelium (8). HPV-associated cervical lesion progression involves sustained viral oncogene expression primarily caused by viral genome integration into host cells. It also entails accumulation of genetic alterations, immune evasion, inflammation, and metabolic reprogramming (9–11). Oxidative stress (OS) is related to both inflammation and metabolic reprograming, and its products, reactive oxygen and nitrogen species, can damage biomolecules, including DNA, leading to genomic instability (12). For example, OS can stabilize HIF-1α, increasing glycolytic metabolism and angiogenic events (11). Since epidemiological and clinical data support that LSIL and HSIL are distinct clinical entities, and because inflammation and OS are important in carcinogenesis, we aimed to characterize the molecular differences between LSIL and HSIL to identify progression markers and therapeutic targets. We initially screened inflammation and OS related mRNA levels in LSIL and HSIL samples. Based on these results, we selected two target genes involved in OS and inflammation, UCP2, uncoupling protein 2 (13, 14), and STAT1, signal transducer and activator of transcription 1 (15, 16). Separately, these genes ‘ products display roles in carcinogenesis, although data is scarce for their role in cervical carcinogenesis. Interestingly, in cardiac hypertrophy, STAT1 displays a protective role, mediated by UCP2/P-DRIP1 (dynamin-related protein-1) induction of mitochondrial fission and increased activity (17). As tumor cells are constantly under selective pression, we rationalized that these proteins might be coexpressed also in cancer and play a role in cervical cancer progression. Therefore, besides gene expression, we also evaluated protein expression in immortalized and cervical cancer-derived cell lines and in precursor lesions and cervical cancer biopsies. One motivation for this work was to find markers that could aid in patient follow-up management in public health services. Exploring these molecules’ expression patterns could help clinicians define the care plan for patients with precursor lesions and may also point to therapeutic targets for cervical cancer.

## 2 Materials and methods

### 2.1 Patients

This study was approved by the Institute of Biomedical Sciences Ethics Committee and the Ethics Committee for Research at the Hospital das Clínicas, School of Medicine, Universidade de São Paulo (National Research Ethics Committee projects 03375412.4.0000.5467 cohort 1; 82664418.5.3001.0068 cohort 2; 03350012.2.0000.0065 cohort 3). All patients signed an informed consent form before sample collection. All methods adhered to the guidelines established by CONEP, the Brazilian National Research Ethics Committee.

Cohort 1 included 16 patients with clinical indications of cervical lesions referred to the Hospital das Clínicas, Universidade de São Paulo, or Hospital Heliópolis (**Table 1**). Diagnosis was confirmed by colposcopy and histology. Lesion fragments were used for RNA extraction and gene expression profiling studies. Exclusion criteria included pregnancy, immunosuppression, immunodeficiency, and other comorbidities. Inclusion required LSIL or HSIL diagnosis. Patients had not undergone prior treatment for cervical lesions. Liquid-based cytology samples from all patients were used for HPV genotyping assays. A total of 8 LSIL and 8 HSIL fragments were collected. However, adequate RNA was successfully isolated from 4 HSIL and 3 LSIL samples for gene expression analysis.

**Table 1:** Epidemiological and clinical data from patients selected for RNA expression studies.

Parameters	Variables	Number (%)	Significance
Total number of patients		16 (100)	
Menopaused subjects	yes	01 (6.25%)	p<0.05
	no	15 (93.75%)	
Smokers	yes	01 (6.25%)	p<0.05
	no	15 (93.75%)	
Sexually active	yes	14 (87.5%)	p<0.05
	no	02 (12.5%)	
Sexual partners	1	01 (6.25%)	p<0.05
	2-4	3 (18.75%)	
	5+	12 (75.0%)	
Previous conization	yes	0	p<0.05
	no	16 (100%)	
Hormonal contraception	yes	7 (43.75%)	
	no	9 (56.25%)	
Delivery	nulliparous	5 (31.25%)	
	1	3 (18.75%)	
	2-4	8 (50.0%)	
HPV genotype	*LSIL*	*7 (43.7%)*	
	HPV16	0	
	HPV18	0	
	other types	5 (71.4%)	
	multiple infection^a^	2 (28.6%)	
	*HSIL*	*9 (56.3%)*	
	HPV16	3 (33.3%)	
	HPV18	0	
	other types	4 (44.4%)	
	multiple infection	0	
	n.d.	2(22.2%)	

a both multiple infection samples included HPV16. The HPV genotype category was subdivided into LSIL and HSIL, with frequencies for specific types within each of the categories. p-values indicate differences between categories in each parameter described, determined by chi-square.

Cohort 2 involved 20 patients with clinical indications of cervical lesions referred to the Hospital das Clínicas, Universidade de São Paulo. This cohort was previously described by Alvarez and colleagues (18). Briefly, these patients were diagnosed with cervicitis, low- and high-grade intraepithelial neoplasia, and invasive cancer. Although the cancer group was older, the age difference was not statistically significant. Most lesions exhibited high-risk HPV detection (65% in cervicitis, 83% in LSIL, 92% in HSIL, and 95% in invasive cancer). There were no significant differences in the percentage of smokers across groups. However, more women were menopausal in the cervical cancer group (28% vs. none in cervicitis, 10% in LSIL, and 4% in HSIL). None had prior treatment for the cervical lesion. The samples included formalin-fixed, paraffin-embedded biopsies from 5 cases of cervicitis, 5 cases of LSIL, 5 cases of HSIL, and 5 cases of invasive carcinomas. Inclusion criteria were age above 18 years old and indication of cervical biopsy for intraepithelial neoplasia or cancer suspicion. Exclusion criteria included pregnancy, immunosuppression, immunodeficiency, and other comorbidities.

Cohort 3 involved patients with cervical cancer referred to the Instituto do Câncer do Estado de São Paulo for treatment. This was a retrospective study with patients enrolled at ICESP for cervical cancer treatment, with a 5-year follow-up. Tissue samples were stored at the Pathology Service, and patient data were available from the RedCAP biorepository. A total of 88 samples were organized into a tissue microarray (TMA). Inclusion criteria were a diagnosis of squamous cell carcinoma (SCC) or adenocarcinoma (ADC) and available tissue from the primary tumor. Exclusion criteria included other tumor types besides SCC or ADC. Patient data are summarized in **Table 2** (19).

**Table 2:** Epidemiological and clinical data from patients selected for the TMA.

Parameters	Variables	Number (%)	Significance
Histologic type	SCC	77 (87.5)	p<0.05
	AC	11 (12.5)	
FIGO classification	1A1	2 (2.3)	p<0.05
	1B1	1 (1.1)	
	1B2	2 (2.3)	
	1B3	3 (3.4)	
	2A	2 (2.3)	
	2B	15 (17.0)	
	3A 3B	2 (2.3) 4 (4.5)	
	3C1	35 (39.8)	
	3C2	10 (11.4)	
	4A	12 (13.6)	
HPV genotype	HPV16	32 (36.4)	p<0.05
	HPV18	5 (5.7)	
	Other high-risk	26 (29.5)	
	Multiple infection	5 (5.7)	
	HPV negative	15 (17)	
	Not determined	5 (5.7)	
Recurrence	Yes	61 (69.3)	p<0.05
	*Local*	*4 (4.5)*	
	*Regional*	*22 (25.0)*	
	*Distant*	*35 (39.8)*	
	No recurrence	25 (28.4)	
	Unknown	2 (2.3)	
Treatment of locally advanced cervical cancer (78 patients)	Radiotherapy only	20 (25.6%)	p<0.05
	Definite chemoradiation	58 (74.4%)	
5-years Disease Free Survival	Yes	21 (23.9)	p<0.05
	No	65 (73.9)	
	Unknown	2 (2.3)	
Tobacco smoker	No	41 (46.6)	
	Yes	44 (50.0)	
	n.d.	3 (3.4)	
Menopause	Yes	44 (50.0)	
	No	44 (50.0)	
STAT1 expression	Yes	56 (63.6)	p<0.05
	*1 nucleus/cytoplasm*	*20 (35.7)*	
	*2 cytoplasm*	*33 (58.9)*	
	*3 nucleus*	*3 (5.4)*	
	No	26 (29.5)	
	n.d.	6 (6.8)	
UCP2 expression	Yes	47 (53.4)	p<0.05
	*1 nucleus/cytoplasm*	*11 (23.4)*	
	*2 cytoplasm*	*32 (68.1)*	
	*3 nucleus*	*4 (8.5)*	
	No	34 (38.6)	
	n.d.	7 (8.0)	

p-values indicate differences between categories in each parameter described, determined by chi-square.

### 2.2 Gene expression assay

To compare gene expression patterns between LSIL and HSIL samples, biopsies were finely minced on a dish kept on ice. RNA was extracted using Trizol reagent (Life Technologies, Carlsbad, CA, US) following the manufacturer’s instructions. RNA concentration and purity were assessed with the ND-1000 Spectrophotometer (NanoDrop Technologies, Thermo Scientific, Waltham, MA). Samples with an absorbance ratio at 260nm/280nm less than 1.8 were discarded. RNA samples were then treated with RNase-free DNase I (Thermo Scientific, Waltham, MA) according to the manufacturer’s instructions. cDNAs were synthesized using the RevertAid Minus First Strand cDNA Synthesis Kit (Thermo Scientific, Waltham, MA). Quantitative RT-PCR assays were performed with the Oxidative Stress RT2 Profiler PCR and the Innate and Adaptive Immune Response RT2 Profiler PCR (PAHS-065YC and PAHS-052ZA, Qiagen, Germany). This system includes RNA quality control checks. The expression levels of target genes were compared to housekeeping genes (GAPDH-Glyceraldehyde-3-Phosphate Dehydrogenase, and ACTB-Actin Beta) using the Livak and Schmittgen method (20). Data analysis was carried out with Qiagen’s platform (https://geneglobe.qiagen.com/br), employing Student’s t-test to compare gene expression between LSIL and HSIL samples. Although data from the Qiagen software are presented, we confirmed the statistical differences using the non-parametric Mann-Whitney U test on the ΔCt values between HSIL and LSIL samples. Notably, by using cervical biopsies, we included both epithelial and stromal compartments in our analyses.

### 2.3 HPV genotyping

HPV genotyping was previously conducted for cohorts 2 (18) and 3 (19). In this study, we collected liquid-based cytology samples from cohort 1 patients for DNA extraction and HPV genotyping. DNA was purified through digestion with proteinase K and phenol-chloroform-ethanol extraction. The DNA concentration was measured using an ND-1000 Spectrophotometer (NanoDrop Technologies – Thermo Scientific, Waltham, MA). Samples with an absorbance ratio at 260nm/280nm less than 1.8 were excluded. DNA integrity was further verified by PCR detection of the human Beta-Globin gene. PCR was performed using the PGMY09/11 primers for HPV DNA detection (21). HPV-positive samples were then hybridized against specific targets using the HPV Linear Array kit (Roche Molecular Diagnostics, Alameda, CA), which can differentiate 37 low- or high-risk HPV genotypes.

### 2.4 Cell lines and cell culture

HaCaT (CVCL_0038) is a spontaneously immortalized human keratinocyte cell line (22) that was transduced in our laboratory with the pLXSN retroviral vector containing HPV16 E6 and E7 oncogenes to generate the cell line HaCaT E6E7. Additionally, HaCaT cells were transduced with the empty vector to produce HaCaT pLXSN. SiHa (CVCL_0032) is a cervical cancer-derived cell line that contains integrated copies of the HPV16 genome and expresses E6 and E7 (23). Cells were maintained in low-glucose Dulbecco’s Modified Eagle’s Medium, DMEM (Thermo Scientific, Carlsbad, CA), supplemented with 10% fetal bovine serum (FBS, Cultilab, Brazil), 1 mg/ml gentamicin (Thermo Scientific, Carlsbad, CA), and 2 g/l sodium bicarbonate at 37 °C in a 5% CO2 atmosphere. For immunofluorescence assays, cells were seeded on glass coverslips pre-treated for 30 minutes with 30% FBS in PBS. After 24 hours, coverslips were transferred to PBS, and cells were washed twice before fixation. For organotypic cultures, 2×10^5 newborn foreskin human keratinocytes (PHKs; Lonza, CH) were seeded onto a 3 mg/ml collagen I (Sigma-Aldrich, St. Louis, MO) bed containing 10^6 J2 fibroblasts and exposed to air/medium (KGM Gold, Lonza, CH) conditions for 12 to 14 days at 37 °C in a 5% CO_2_ atmosphere. Finally, organotypic cultures were carefully fixed in buffered formaldehyde, embedded in paraffin, and sectioned crosswise in 4 μm slices for immunofluorescence or immunohistochemistry.

### 2.5 Protein expression assays

The expression levels of UCP2 and STAT1 in samples from cohorts 2 and 3, along with organotypic cultures, were assessed by immunohistochemistry or immunofluorescence. Paraffin-embedded tissue sections, 4 μm thick, from biopsies or organotypic cultures, were treated with xylol and rehydrated through an ethanol gradient from 100% to 50%. Antigen retrieval involved incubating the tissues in boiling sodium citrate solution at pH 8.0 for 10 minutes. Peroxidase activity was quenched with Bloxall reagent (Vector Laboratories, Newark, CA). Primary antibodies used included rabbit anti- human UCP2 (ab97931, Abcam, Cambridge, MA, UK) at a 1:150 dilution and rabbit anti-human STAT1 antibody [EPRR 21057- 168] (ab 210524, Abcam, Cambridge, MA, UK) at a 1:1000 dilution, applied to pre- blocked tissues in 5% FBS and 0.5% Tween 20 in PBS. For immunohistochemistry, the ImmPRESS Universal kit (Vector Laboratories, Newark, CA) was employed, with detection using DAB (3,3’- diaminobenzidine) substrate kit. Tissues were counterstained with hematoxylin before mounting with Permount (Sigma- Aldrich, St. Louis, MO). To determine if STAT 1 and UCP 2 expression could be regulated by HPV oncoproteins, an immunofluorescence assay was performed on 2D and 3D HaCaT cultures transduced with the pLXSN retroviral vector containing HPV 16 E6 and E7 oncogenes. For immunofluorescence, organotypic cultures were processed as described up to the antigen retrieval step. Cells on coverslips were fixed in 100% methanol for 5 minutes and washed three times in PBS. Subsequently, both organotypic cultures and cells on coverslips were treated similarly. After blocking, samples were incubated with the primary antibodies at the dilutions specified above. Following washing, cells or tissues were incubated with secondary anti-rabbit IgG Alexa 488 (Cell Signaling Technology, Danvers, MA) diluted 1: 1, 000 for 30 minutes at room temperature. After final washes, samples were mounted with Fluorshield DAPI (Sigma- Aldrich, St. Louis, MO). Images were captured using an Olympus BX 61 microscope with a DC 70 camera and Olympus Life Science software (Japan). Quantification was performed with FIJI software.

### 2.6 Statistical analyses

Results are presented as means and standard deviations. Pearson correlation and ANOVA analyses were performed using JASP software, JASP team (2023) JASP (version 0.17.3). Tukey’s *post-hoc* test was performed to compared different experimental groups tested by ANOVA. Binary correlations and Kaplan-Meier curves were conducted using SPSS software (IBM, Armonk, NY). Differences between experimental groups were considered significant when the p-value was below 0.05.

## 3 Results

### 3.1 Inflammatory and oxidative stress-related gene expression increases with cervical lesion grade

Inflammation and oxidative stress play a significant role in carcinogenesis; therefore, we examined the expression of genes associated with both processes by comparing LSIL and HSIL biopsies (cohort 1). Patients with LSIL and HSIL had similar mean ages: 38.2 years old in the LSIL group (range: 27 to 47 years) and 37.7 years old in the HSIL group (range: 25 to 46 years). Most were sexually active (87.5%) and reported having more than five sexual partners in their lifetime (75%). Half of the patients had between 2 and 4 deliveries, and 31.5% were nulliparous at the time of the study (**Table 1**). When comparing LSIL and HSIL mRNA expression, among all 82 targets available in the Qiagen platform, we found significant upregulation of eight genes related to inflammation and five ones related to oxidative stress, some of which are known to be regulated in HPV-infected or transformed cells (inflammation: STAT3 (24), MAPK1, STAT1 (25)], MX1, IFNGR1, STAT6, MYD88, NFKB1A; oxidative stress: FOXM1 (26), PRDX2 (27), NCF2, UCP2, and TNX) (**Table 3**). Additionally, we observed a downregulation of nine genes involved in inflammatory responses: CD40L, CD8A, CXCR3, IL-1α, IL-2, ITGAM, TNFα, TLR7 (28), and TNFR, although these differences did not reach statistical significance. Several of the upregulated genes are important in cancer biology, including cervical cancer, such as STAT3 (24), NFKB1A (29), and PRDX2 (27). Among the differentially expressed genes, we identified the IFNGR/STAT1 axis, including the STAT1 target MX1 (30). We selected STAT1 and UCP2 for validation and further investigation.

**Table 3:** Differentially expressed genes between LSIL and HSIL.

Gene	Fold change	P value
STAT3	4.29	0.044
MAPK1	5.18	0.019
*STAT1*	4.27	0.046
*MX1*	10.98	0.002
*IFNGR1*	4.41	0.006
STAT6	7.28	0.005
MYD88	4.94	0.029
NFKB1A	2.8	0.040
FOXM1	3.73	0.048
PRDX2	3.2	0.024
NCF2	2.51	0.009
UCP2	2.13	0.042
TNX	4.48	0.023

### 3.2 HPV oncoproteins control STAT1 and UCP2 expression

**Figure 1:**
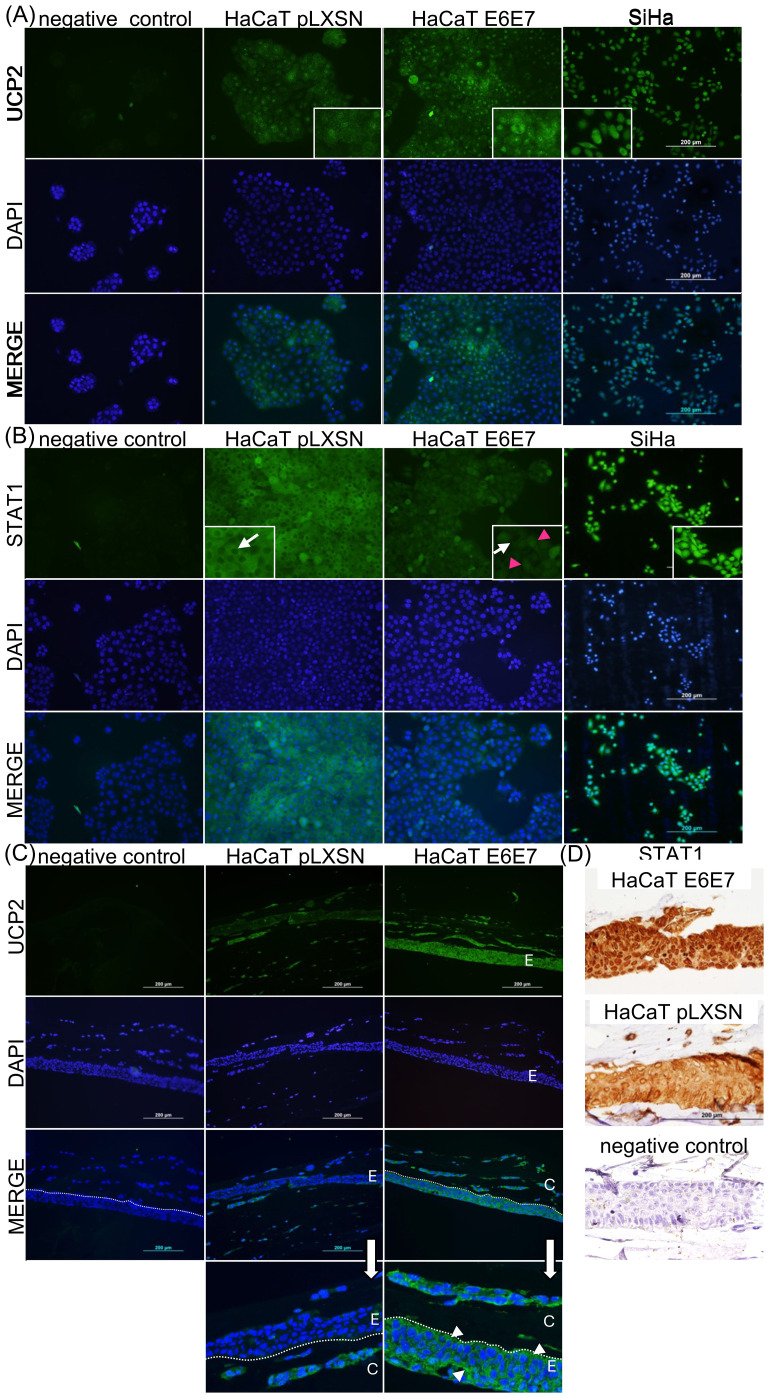
HPV oncoproteins’ expression is linked to increased UCP2 levels and STAT1’s cellular location. UCP2 **(A)** and **(C)** and STAT1 **(B)** and **(D)** were detected through immunostaining in 2D **(A, B)** and 3D **(C, D)** cell cultures. The indicated cell lines were grown on glass coverslips or seeded on a collagen I scaffold and cultured for 12 days prior to harvesting. UCP2 and STAT1 were identified using specific primary antibodies, followed by a secondary antibody conjugated with Alexa 488 (green). Cells were counterstained with DAPI (blue). In **(C)**, dotted lines mark the epithelial basal layer; E indicates the epithelium, and C the collagen layer. In **(D)**, anti-STAT1 primary antibody was detected through the ImmPress polymer and DAB reaction with peroxidase, resulting in a brown precipitate. Insets in **(A)**, **(B)** provide higher magnification images to show UCP2 and STAT1 details. In **(B)**, white arrows point to cytoplasmic STAT1 expression, with a negative nucleus in HaCaT pLXSN cells, while the red arrow highlights a positive nucleus in the HaCaT E6E7 cell line. In **(C)**, white arrows indicate areas of higher magnification for detailed observation and arrowheads indicate cytoplasmic UCP2 localization. Negative controls involved the same cells or 3D cultures processed identically but without primary antibody incubation. Images were acquired using a BX61 Olympus microscope, with an attached DC70 camera and associated software.

First, we investigated whether HPV oncoproteins could regulate the expression of STAT1 and UCP2. We compared their levels using immunofluorescence or immunohistochemistry in both 2D and 3D cultures. These cultures involved the immortalized keratinocyte cell line HaCaT (22), transduced with HPV16 E6 and E7 oncogenes (HaCaT E6E7), or a control cell line with an empty vector (HaCaT pLXSN), along with the SiHa cell line (**Figure 1**). In 2D cultures, we observed positive, but low UCP2 expression in HaCaT pLXSN cells, which increased in HaCaT E6E7 (2.5 ± 1.2-fold change compared to the control) (**Figure 1A**). Meanwhile, STAT1 expression was higher in the HaCaT pLXSN than in HaCaT E6E7 (0.47 ± 0.6-fold change from control to E6E7 expressing cells; **Figure 1B**). Interestingly, we also observed differences in protein localization. UCP2 expression was mainly localized in puncta distributed through the cell, indicating mitochondrial localization. STAT1, however, was cytoplasmic in HaCaT pLXSN cells, but in HaCaT E6E7, we found mostly nuclear localization, with a 3.37-fold increase in positively labeled nuclei compared to the control (white arrows indicate negative nuclei, red arrows indicate positive nuclei). SiHa cells displayed high UCP2 and STAT1 expression. UCP2 was mainly cytoplasmic in this cell line, while STAT1 localized in the nuclear and cytoplasmic compartments (**Figure 1B**). In 3D cultures, tissue organization affected the expression of both proteins. HaCaT E6E7 cells displayed significantly higher levels of UCP2 and STAT1 than HaCaT pLXSN cells (**Figures 1C, D**). UCP2 expression was cytoplasmic (**Figure 1C**, arrows). STAT1 translocated to the nuclei in HaCaT E6E7 cells, with a 13.3-fold increase in the percentage of labeled nuclei (black arrows) (**Figure 1D**).

### 3.3 UCP2 and STAT1 protein expression are upregulated during precursor cervical lesion progression

**Figure 2:**
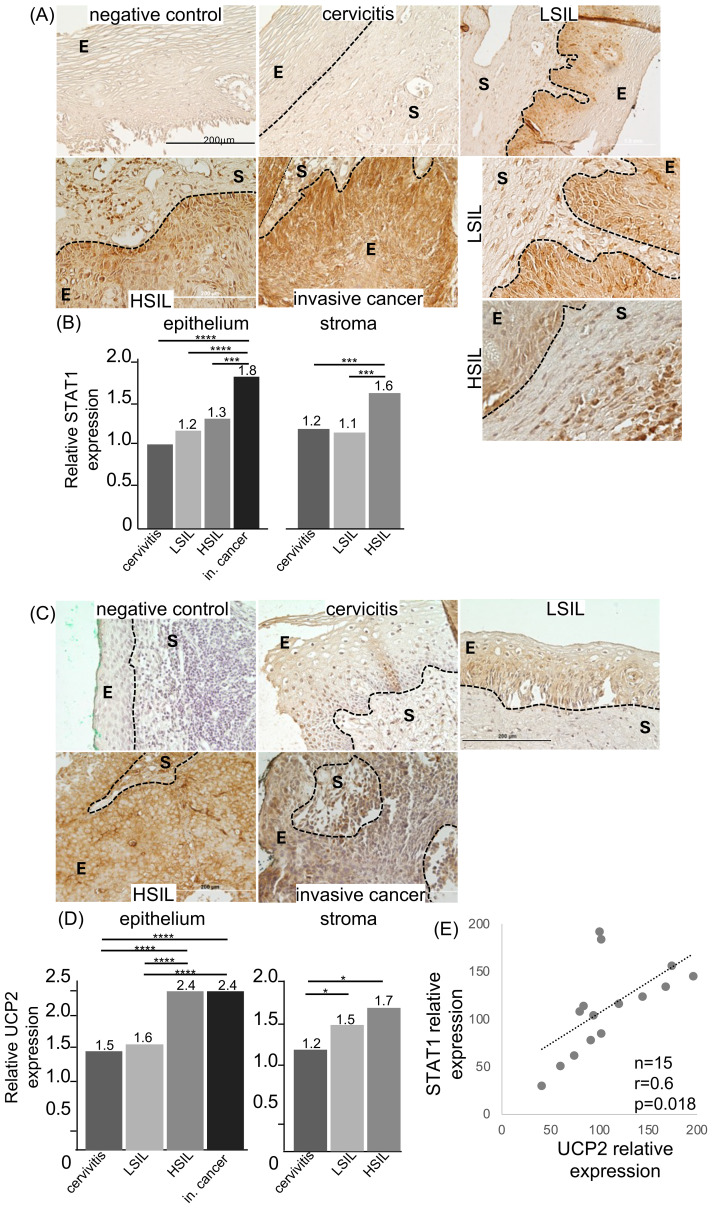
STAT1 and UCP2 Expression Increase as Cervical Lesions Progress. The levels of STAT1 and UCP2 were measured by immunohistochemistry in a set of cervical samples with lesions of various grades and cancer. **(A, C)** show representative immunohistochemistry images from lesions of specified grades using anti-STAT1 **(A)** and anti-UCP2 **(C)** antibodies. The epithelial and stromal areas are labeled as E and S, respectively. The boundary between these areas is marked with a hatched line. In A, on the right side, images of LSIL and HSIL are shown at higher magnification. **(B, D)** display the quantification of STAT1 and UCP2 expression. Relative expression was calculated as: negative control means densitometry values divided by cervicitis/LSIL/HSIL, and Invasive Carcinoma I (in carcinoma) means densitometry. ANOVA analysis was performed using JASP software. Significant differences between experimental conditions are indicated above histograms: p<0.5 *, p<0.0001 ***, p<0.00001 ****. The values in the histograms represent the fold change in expression for each condition compared to cervicitis in the epithelial compartment. **(E)** Shows the Pearson correlation between STAT1 and UCP2 expression. In all cases, five biopsies from each lesion or cancer type (invasive cancer or In. cancer) were analyzed. Images were captured with a BX61 Olympus microscope and a DC70 camera (five fields per biopsy). Image data analysis was conducted using FIJI software.

We then aimed to validate the RNA expression data through immunohistochemistry using antibodies against UCP2 and STAT1 in 20 cervical precursor lesions (5 cervicitis, 5 LSIL, 5 HSIL) and 5 cervical cancer samples from 20 patients in a previously described cohort (cohort 2) (19). We observed that STAT1 expression increased with lesion grade, reaching its highest in invasive cancer samples (**Figures 2A, B**). Importantly, we noted an increase in STAT1 expression in both the epithelial and stromal compartments. In the epithelial compartment, STAT1 was detected in both the cytoplasm and nucleus (details on the right side of **Figures 2A–E**: epithelium, S: stroma). In the stromal compartment, there was a sharp increase in STAT1 expression from LSIL to HSIL, with strong nuclear staining, suggesting this pathway may be active in HSIL stromal cells and possibly reflecting increased cellular infiltration in these lesions (18). In cancer samples, the stromal compartment was barely represented, preventing further analysis. Our data showed a strong positive correlation between lesion grade and STAT1 expression within the epithelial compartment (Pearson r=0.855, p=0.0008), but not in the stromal compartment. We observed a significant increase in UCP2 expression in both epithelial and stromal areas as lesions progressed to cancer. However, UCP2 expression peaked in HSIL samples and remained high in invasive cancer samples (**Figures 2C, D**). UCP2 was primarily cytoplasmic in most samples, although nuclear expression was observed in some invasive cancer cells. In the stromal compartment, UCP2 expression increased with lesion grade, although low representation prevented evaluation in the stromal areas of invasive cancer samples. A positive correlation was found between UCP2 expression and lesion progression in the epithelial compartment (Pearson correlation r=0.65, p=0.0017). Notably, we also found a significant positive correlation between STAT1 and UCP2 levels in these samples (**Figure 2E**).

### 3.4 UCP2 positive expression in cancer correlates with better prognosis

Finally, we examined whether STAT1 and UCP2 expression could have prognostic value for cancer patients. To do this, we studied a cohort of cervical cancer patients (19), cohort 3, whose samples were organized into a tissue microarray (**Table 2**). This cohort included 88 patients with a mean age of 51.0± 14.1 years. These patients had a higher parity rate compared to the Brazilian average, with 4.47± 3.43 deliveries per patient. Half of the patients reported smoking cigarettes. Additionally, 50% of the patients were postmenopausal by the time invasive cancer was diagnosed. Most patients presented with FIGO stage III cancer at diagnosis (stage I 9.1%, stage II 19.3%, stage III 58%, and stage IV 13.6%). Local and distant recurrences occurred in 61 patients (69,3%), with a 5-year disease-free survival rate of 23.9%. HPV16 was the most prevalent HPV type (32 as single infections, plus 4 of the multiple infections, corresponding to a total of 40.9% of the samples), while other high-risk oncogenic HPV types were detected in 36.4% of the samples that included the following samples: 5 HPV18, 26 other high-risk types and 1 multiple infection without HPV16. There were 15 (17%) HPV negative samples, all positive for β-Globin, and 5.7% of the samples were not genotyped (**Table 2**). In this cohort, patients with locally advanced cervical cancer (LACC), FIGO stages IIB, III, or IV, had worse survival outcomes (p=0.027). Due to the advanced stage of disease in our patients, standard treatment consisted of definitive chemoradiotherapy (**Table 2**).

**Figure 3:**
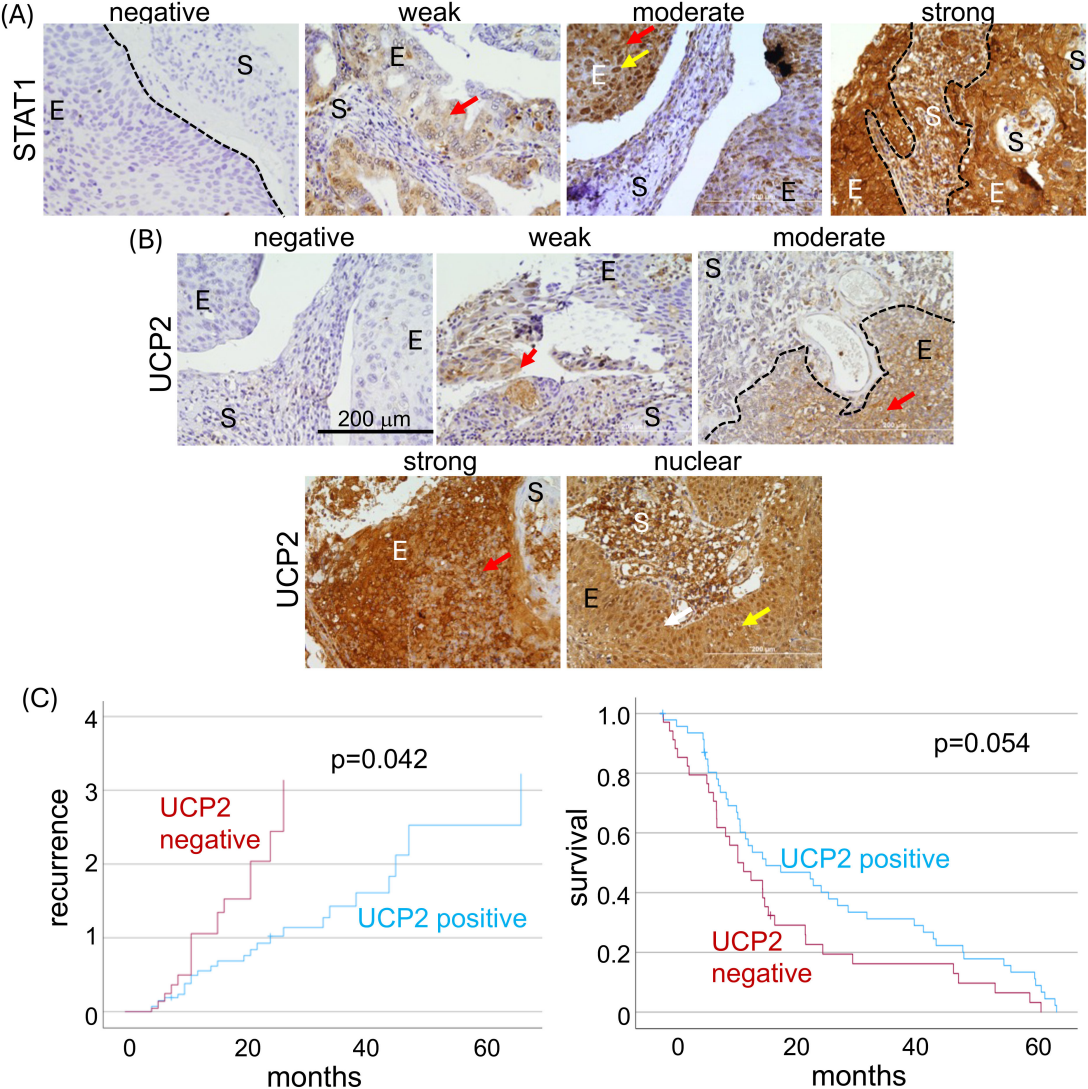
UCP2 expression is associated with lower recurrence rates and higher overall survival rates in cervical cancer patients. The levels of UCP2 were measured using immunohistochemistry in a collection of cervical cancer samples embedded in a tissue microarray. Representative images from these samples stained with anti-STAT1 **(A)** and anti-UCP2 **(B)** antibodies are shown. The tissues on the microarray were categorized based on signal intensity and cellular localization. Signal intensity was classified as: negative (fewer than 10% of cells labeled), weak (10 to 50%), moderate (50 to 80%), and strong (more than 80%). Localization was identified as negative, nuclear (indicated by a yellow arrow), cytoplasmic (red arrow), or both nuclear and cytoplasmic. The stromal and epithelial compartments are marked as S and E, respectively. Images were taken with a BX61 Olympus microscope equipped with a DC70 camera and appropriate software. **(C)** shows the patient’s recurrence risk associated with UCP2 expression (left graph) and the patient’s cancer-specific survival based on overall UCP2 expression (right graph). p-values are noted on each graph, and the follow-up period is given in years. .

We assessed STAT1 and UCP2 protein expression by immunohistochemistry in cohort 3. The expression levels were categorized as negative, weak, moderate, or strong. Weak staining indicated samples with 10% to 50% positively labeled cells; moderate, 50% to 80%; and strong, more than 80% positively labeled cells (**Figures 3A, B**). We grouped strong and moderate expression as positive, and weak as negative. Additionally, we classified expression based on subcellular localization: nuclear, cytoplasmic, or both. STAT1 was positive in 63.6% of samples, negative in 29.5%, and 6.8% were lost during processing. Among positive samples, 35.7% showed both nuclear and cytoplasmic expression, 58.9% only cytoplasmic, and 5.4% only nuclear. UCP2 was detected in 53.4% of samples (positive), with 23.4% exhibiting both nuclear and cytoplasmic, 68.1% only cytoplasmic, and 8.5% only nuclear expression (**Table 2**). For UCP2 immunohistochemistry, 7.9% of samples were lost, and 38.6% showed weak or no protein expression. Most UCP2 expression was cytoplasmic, with only four exceptions showing nuclear antibody reaction. A weak but significant positive correlation was observed between UCP2 and STAT1 expression (r=0.281, p=0.012). Notably, adenocarcinomas were more likely to express both proteins (p=0.019), whereas cervical squamous carcinomas showed an equal distribution of samples positive and negative for both or each of the proteins and other expression patterns.

Overall, STAT1 expression did not correlate with disease recurrence. In contrast, UCP2 positive expression showed a negative association with poor prognosis in cancer patients. Among patients with recurrence, 50% of the samples had moderate or strong UCP2 expression (positive), while the remaining 50% had weak or negative expression (negative). In patients without recurrence, 76% of the samples displayed positive UCP2 expression, whereas 24% showed negative expression (p=0.066). Over time, UCP2 positive expression was associated with improved patient survival (p=0.054) (**Figure 3C**). Additionally, UCP2 positive expression correlated with a lower risk of recurrence (p=0.042) (**Figure 3C**). No correlations were observed between treatment and UCP2 or STAT1 expression. Lastly, a positive correlation was found between smoking and STAT1 expression (Pearson correlation r=0.283, p=0.010), but no other clinical parameters were associated with STAT1 or UCP2 expression.

## 4 Discussion

HPV is the etiologic factor in cervical cancer development. In productive infections, the viral genome is primarily episomal. However, during persistent infections, the likelihood of viral integration into the host genome increases, potentially leading to higher and constitutive expression of E6 and E7 oncogenes. E6 and E7 are pleiotropic proteins responsible for the immortalization and transformation of infected epithelial cells, disrupting several signaling pathways (5, 6).

In this study, we showed that UCP2 and STAT1 expression are affected by the E6 and E7 proteins expressed in the keratinocyte cell line HaCaT. Notably, we observed more pronounced results in 3D cultures compared to monolayers. The cell organization influenced the expression of both STAT1 and UCP2. The most significant differences between E6/E7-expressing cells and controls were observed in UCP2 expression and STAT1 localization in organotypic cultures. STAT1 nuclear localization indicates activation, as it is a transcription factor. A study by Hong and collaborators (31) reported that HPV16 and HPV31 suppress STAT1 expression in both monolayer cells and organotypic cultures. Our findings align with theirs in monolayer cultures, but differ in organotypic cultures derived from HaCaT cells expressing HPV16 E6E7, which showed a notable increase in STAT1 levels. This variation may be attributed to the experimental model, as we used an immortalized cell line, whereas Hong and collaborators (31) used primary human keratinocytes. Additionally, a strong viral promoter, LTR, drove E6 and E7 expression in the HaCaT cells, another difference between the models. Nonetheless, our data suggest that HPV can modulate STAT1 expression. Aberrant STAT1 expression has been linked to cancer biology, serving as both a prognostic marker and a target for cancer therapy (32–34). Interestingly, Yi and colleagues, using single-cell RNA sequencing and bioinformatic tools, were able to divide cervical carcinogenesis into a stepwise process involving four gene hubs (35). STAT1 expression was present in all gene hubs, with particular emphasis on the fourth, which represents the transition from HSIL to invasive cancer. STAT1 is a transcription factor activated by both type I and type II Interferons. However, data also show interferon-independent activation of STAT1. For example, during viral infections, it can be activated via the cGAS/STING pathway in a SKY-dependent manner. Interestingly, a study by Qiao and colleagues (36) showed that SiHa cells express both cGAS and STING and can have this pathway activated by intracellular DAMPs (cellular damage-associated molecular patterns). In our work, we observed nuclear localization of STAT1 in biopsies, as well as in tumor cells and HaCaT cells transduced with E6 and E7. The mechanism behind this remains unknown, but tumor cells may accumulate DNA fragments in the cytoplasm, activating the cGAS/STING pathway and subsequently STAT1. It is well established that HPV infection causes genomic instability and can lead to the accumulation of DNA fragments in the cytoplasm (37). Of particular interest, there is evidence that STAT1 can promote radiosensitivity by regulating PARP1 [Poly (ADP-ribose) polymerase-1] expression (34). Still, controversy exists in this field, with reports indicating both positive (38) and negative (39) correlations between STAT1 expression and cancer progression. Our data support that STAT1 is upregulated as cervical lesions progress to cancer and suggest that STAT1 may serve not only as a prognostic marker for lesion development but also as a therapeutic target. However, our data also shows that in cancer patients, STAT1 levels do not correlate with clinical parameters.

Several cancer types show UCP2 overexpression (40). UCP2 belongs to the mitochondrial uncoupling protein family and functions as a metabolic hub mediating pyruvate transport (41) and glutamine metabolism (42). Additionally, UCP2 influences cell growth and movement in endothelial cells by reducing ROS, which prevents p53 activation and may aid in carcinogenesis (43, 44). There is limited data on UCP2’s potential role in cervical cancer and virtually none regarding precursor lesions. UCP2 knockdown has been shown to arrest the proliferation of cervical cancer-derived cells (45). Conversely, its expression seems to predict a better response to treatment (46, 47). High-risk HPV E6 protein can indirectly affect UCP2 levels. E6 causes p53 degradation via the proteasome and can suppress miR-34a, leading to increased UCP2 expression (48). Likewise, p65 NF-κB can boost miR-2909, which inhibits KLF-4, a factor that normally suppresses UCP2 (49). Importantly, evidence suggests HPV oncoproteins trigger p65 NF-κB activation (24). This may partly explain UCP2 levels in SiHa and HaCaT E6E7 cells compared to controls. Conversely, UCP2 may also activate the NF-κB pathway (40). Currently, we can only speculate why patients with UCP2-positive tumors tend to have better outcomes. However, data show antigen stimulation increases UCP2, which is crucial for anti-tumor responses by CD8 T cells (50). This could explain why UCP2-positive tumors are linked to improved prognosis. Although UCP2 expression in the stromal tissue of our samples was not quantified, we observed positive UCP2 staining in stromal areas of precancerous lesions and cancer. Our findings suggest UCP2 should be considered in cervical cancer, not just as a prognostic marker but also as an indicator of treatment response, especially for patients undergoing immunotherapy.

We need to comment on the use of different cohorts and experimental models in this work. We did not have access to tissue from the biopsies used in the RNA expression profile experiments and had to use other samples for data validation. It would have been better to use the same samples for both RNA and protein expression experiments, and then a different cohort for validation. Unfortunately, this was not possible. However, we are confident the data is solid, and the cell lines we used helped confirm our results.

We have not addressed, in this work, STAT1 and UCP2 function in transformed cells. We are, at the moment, conducting experiments to downregulate the expression of each of these proteins to study their role in cancer cells and if there is a mechanism linking them, as well as other targets that we identified as differentially expressed.

In conclusion, our results showed that STAT1 and UCP2 are upregulated in cervical precursor lesions as they progress to cancer. UCP2 upregulation and STAT1 nuclear localization are, at least in part, regulated by HPV16 oncoproteins. Our data suggest that positive UCP2 and STAT1 expression may indicate the transition from LSIL to HSIL, thus warranting attention. In cervical cancer patients, however, UCP2 serves as a marker of positive prognosis.

## Data Availability

The datasets presented in this study can be found in online repositories. The names of the repository/repositories and accession number(s) can be found below: http://repositorio.uspdigital.usp.br/handle/item/719, 2025-01-30.
